# Phenotypic differentiation of the *Solidago virgaurea* complex along an elevational gradient: Insights from a common garden experiment and population genetics

**DOI:** 10.1002/ece3.3252

**Published:** 2017-07-28

**Authors:** Masaaki Hirano, Shota Sakaguchi, Koichi Takahashi

**Affiliations:** ^1^ Graduate School of Science and Technology Shinshu University Matsumoto Japan; ^2^ Graduate School of Human and Environmental Studies Kyoto University Kyoto Japan; ^3^ Department of Biology Faculty of Science Shinshu University Matsumoto Japan; ^4^ Institute of Mountain Science Shinshu University Matsumoto Japan

**Keywords:** genetic variations, microsatellite, morphological variations, phenology, population structure, subspecies

## Abstract

Plant species distributed along wide elevational or latitudinal gradients show phenotypic variation due to their heterogeneous habitats. This study investigated whether phenotypic variation in populations of the *Solidago virgaurea* complex along an elevational gradient is caused by genetic differentiation. A common garden experiment was based on seeds collected from nine populations of the *S*. *virgaurea* complex growing at elevations from 1,597 m to 2,779 m a.s.l. on Mt. Norikura in central Japan. Population genetic analyses with microsatellite markers were used to infer the genetic structure and levels of gene flow between populations. Leaf mass per area was lower, while leaf nitrogen and chlorophyll concentrations were greater for higher elevations at which seeds were originally collected. For reproductive traits, plants derived from higher elevations had larger flower heads on shorter stems and flowering started earlier. These elevational changes in morphology were consistent with the clines in the field, indicating that phenotypic variation along the elevational gradient would have been caused by genetic differentiation. However, population genetic analysis using 16 microsatellite loci suggested an extremely low level of genetic differentiation of neutral genes among the nine populations. Analysis of molecular variance also indicated that most genetic variation was partitioned into individuals within a population, and the genetic differentiation among the populations was not significant. This study suggests that genome regions responsible for adaptive traits may differ among the populations despite the existence of gene flow and that phenotypic variation of the *S*. *virgaurea* complex along the elevational gradient is maintained by strong selection pressure.

## 1 INTRODUCTION

Plant species distributed along wide environmental gradients often show large phenotypic variation reflecting their habitat heterogeneity (Leiblein‐Wild & Tackenberg, 2014; Turesson, 1922; Vitasse, Delzon, Bresson, Michalet, & Kremer, 2009). Intraspecific phenotypic variation is caused by phenotypic plasticity and genetic differentiation (Frei, Ghazoul, Matter, Heggli, & Pluess, 2014; Scheepens, Frei, & Stöcklin, 2010; Scheepens & Stöcklin, 2013; Vitasse et al., 2014). The phenotypic plasticity determines short‐term morphological and physiological responses to environmental fluctuations (Bradshaw, 1965; Castillo et al., 2014; Sultan, 2000). However, different environmental selection pressures may lead to long‐term evolutionary changes in phenotypes adapting to local environments (Linhart & Grant, 1996). As a result, adaptive traits are genetically fixed over generations by selection. Phenotypic variation due to genetic differentiation along environmental gradients often underlies formation of ecotypes or intraspecific taxa, which are at initial stages of speciation (Fukuda, 1974; Tateoka, 1983). Thus, study on geographic variation of phenotypes is important to understand mechanisms of evolution and intraspecific diversity (Endler, 1977). A powerful way to detect genetic‐based phenotypic differentiation is common garden experiments, which have been applied to many plant species (Bertel, Buchner, Schonswetter, Frajman, & Neuner, 2016; Clausen, Keck, & Heisey, 1948; Kawano, 1974; Kruckeberg, 1967; Scheepens & Stöcklin, 2013; Vitasse et al., 2014).

Mountain ecosystems are ideal to study adaptive differentiation of plant species, because phenotypic variation is often found along elevational gradients, accompanied by drastic changes of environmental conditions at short geographic distances. Temperature and air pressure are lower at higher elevations, while wind velocity and ultraviolet radiation are greater at higher elevations (Friend & Woodward, 1990). Plant species respond to these elevational changes based on their morphological and physiological plasticity and through adaptation. For example, deciduous plant species maintain a positive carbon balance at the level of individual leaves at high elevations with a short growing season by decreasing the leaf construction cost and increasing the assimilative capacity (Kudo, 1996, 1999; Oleksyn et al., 1998). Plant height also generally decreases with increasing elevation because of resource limitation due to severe environmental conditions, that is, short growing season, prolonged snow cover, strong wind, and shallow soil (Clausen et al., 1948; Körner, 2003; Mizuno, 1991; Natori, 1964; Takahashi & Yoshida, 2009). Furthermore, seed traits may change depending on elevation. The germination rate is high in high elevations for certain species (Vera, 1997), and germination and subsequent seedling survival rates are often greater for larger seeds (Jakobsson & Eriksson, 2000; Moles & Westoby, 2004). Therefore, sharp environmental changes along elevational gradients promote differentiation of plant traits to adapt to local environmental conditions. However, genetic differentiation along elevational gradients may be prevented by gene flow because gene flow homogenizes the genetic structure among populations (Lenormand, 2002; Slatkin, 1985). Gene flow is expected to occur along elevational gradients within a mountain because geographic distance is short between populations within a mountain (Matter, Kettle, Ghazoul, & Pluess, 2013). However, even if the elevational range is narrow, differentiation of flowering phenology is caused by the difference in such parameters as timing of snow melting (Kudo, 2000). Nonsynchronous flowering timing among populations along an elevational gradient acts as a barrier against gene flow (Hirao, Kameyama, Ohara, Isagi, & Kudo, 2006). Furthermore, the gene flow in plants with entomophilous flowers along elevational gradients is affected by the activity of pollinating insects (Byars, Parsons, & Hoffmann, 2009). Thus, evaluation of gene flow and genetic structure behind geographic variation of phenotypes is important to improve our understanding of mechanisms of adaptation and evolution of plants.

The *Solidago virgaurea* L. complex (Asteraceae) is a perennial herb species and is widespread in northern Eurasia from the temperate to subarctic zones (Hayashi, 1978a; Makino, 1989). This species grows in various vegetation types from lowlands to the alpine zone (i.e., riverside, forest floor, alpine meadow) and shows large morphological variation. Previous studies reported phenotypic variation of the *S*. *virgaurea* complex along elevational gradients. For example, plant height, leaf mass per area (LMA), and number of flower heads per plant decrease with increasing elevation, while leaf nitrogen and chlorophyll concentrations, diameter of involucres and number of florets per flower head increase with elevation (Nishizawa, Kinoshita, Yakura, & Shimizu, 2001; Sakurai & Takahashi, 2017; Takahashi & Matsuki, 2017).

The *S. virgaurea* complex shows large morphological variation along elevational gradients and so the *S*. *virgaurea* complex in central Japan is classified into two subspecies, a lowland type of *S. virgaurea* L. subsp. *asiatica* Kitam. ex Hara and an alpine type of *S. virgaurea* L. subsp. *leiocarpa* (Benth.) Hultén (Kitamura, 1957; Takasu, 1975). Similarly, two closely related species of the *S. virgaurea* complex, namely *S. virgaurea* s. str. (lowland type) and *S. minuta* (mountain type), are distributed along elevational gradients in Europe (Kiełtyk & Mirek, 2014). Although many previous studies focused on morphological variation of reproductive organs (e.g., shape of involucral scales) to identify the two subspecies in Japan, they did not show whether morphological variation of the *S. virgaurea* complex along elevational gradients was caused by genetic differentiation and adaptation or phenotypic plasticity (Hayashi, 1976, 1978a, 1978b; Kitamura, 1957; Takasu, 1975). The two subspecies of the *S*. *virgaurea* complex collected at nine elevations between 1,260 m and 2,670 m a.s.l. on Mt. Hakusan in central Japan show no differentiation, based on genetic analyses of fluorescence in situ hybridization (FISH) and random amplified polymorphic DNA (RAPD) (Nakamura, Miyamoto, Murata, Yamagishi, & Fukui, 1997). However, the RAPD method may not be suitable to analyze population genetic structure because most of RAPD bands show dominant inheritance and the reproducibility is low (Kagaya, 2005). The shape of involucral scales used to classify the two subspecies continuously changes along elevational gradients and is often intermediate between the two subspecies at the boundary between their distribution areas (Hayashi, 1976, 1977, 1978b; Nakamura et al., 1997). Therefore, the taxonomic treatment of the intraspecific taxa is still controversial. Sakaguchi and Ito (2014) advocated the necessity to examine the population genetic structure and gene flow in populations of the *S. virgaurea* complex.

In this study, we hypothesized that phenotypic variation of the *S. virgaurea* complex along an elevational gradient is caused by genetic differentiation. We made a seed germination experiment and a common garden experiment using seeds collected from nine populations of the *S*. *virgaurea* complex from 1,597 m to 2,779 m a.s.l. in central Japan to confirm the genetic basis of phenotypic variation, and we also analyzed the population genetic structure by using codominant microsatellite markers with high reproducibility and polymorphism. Specifically, we answer the following questions to clarify the hypothesis:

1. Do seed germination traits, plant morphological traits and flowering phenology of the *S*. *virgaurea* complex differ genetically along an elevational gradient?
2. Are the nine populations along the elevational gradient genetically connected to each other by active gene flow?

## 2 MATERIALS AND METHODS

### 2.1 Seed collection

In October 2012, seeds of the *S*. *virgaurea* complex were collected from nine populations at elevations from 1,597 to 2,779 m a.s.l. along an east slope of Mt. Norikura (36°06′N, 137°33′E, 3,026 m a.s.l.) in central Japan (Table 1). At each elevation, seeds were collected from 10 individuals, except for locality at 1,713 m a.s.l. where they were collected from only five individuals (pop_1700, Table 1). Seeds of each individual were put in an envelope and stored at 3°C until the germination experiment. Twenty seeds per individual were weighed, and then, the mean seed weight was calculated for each maternal plant. *S. virgaurea* subsp. *asiatica* and *S. virgaurea* subsp. *leiocarpa* are distributed below and above ca. 1,950 m a.s.l., respectively, on Mt. Norikura, based on morphological characteristics (Nishizawa et al., 2001). Although the lowest sampling site was 1,597 m a.s.l. in this study, individuals at the lowest site were not hybrids between the two subspecies and were typical *S. virgaurea* subsp. *asiatica* (Nishizawa et al., 2001; Takahashi & Matsuki 2017). In addition, as a practical matter, it was difficult to find *S. virgaurea* subsp. *asiatica* in elevations lower than 1,597 m a.s.l. because of human disturbances. Therefore, we could not sample at elevations lower than 1,597 m a.s.l., but our samples included the two subspecies.

**Table 1:** Location of sampling sites of the *Solidago virgaurea* complex populations on Mt. Norikura, central Japan

Population	Elevation (m)	Latitude (N)	Longitude (E)
pop_1600	1,597	36°06′54.34	137°36′46.59
pop_1700	1,713	36°06′31.35	137°36′22.50
pop_1900	1,908	36°06′40.42	137°35′38.67
pop_2000	2,003	36°07′18.48	137°35′18.52
pop_2100	2,109	36°07′25.01	137°34′53.11
pop_2200	2,205	36°07′16.94	137°34′34.85
pop_2300	2,309	36°07′08.60	137°34′21.83
pop_2400	2,406	36°06′59.17	137°34′21.75
pop_2800	2,779	36°06′50.87	137°33′00.14

### 2.2 Germination experiment

A germination experiment was done in April 2013. We used 10 seeds per maternal plant for the experiment (i.e., total of 100 seeds per elevation, except pop_1700 for which 50 seeds were used). Seeds were placed on two filter papers in a petri dish, which were kept wet with de‐ionized water. The petri dishes were placed in an incubator (Hitachi, CRB‐41LA, Tokyo). Temperature and light conditions in the germination experiment were according to Kondo (1990): Temperature was set to 22.5°C throughout the experiment; the daylight duration was 14 hr. Germination was hardly observed after 8th day of the experiment, so the experiment was stopped at 17 day. Germinated individuals were counted every day during the experiment. The germination rate per population was calculated from the number of germinated individuals until the end of the experiment. Germinated individuals were used for the common garden experiment.

### 2.3 Common garden experiment

A common garden experiment was conducted for two growing seasons. In May 2013, germinated individuals obtained in the germination experiment were planted into pots (7.5 cm in diameter) filled with potting compost (Protoleaf Inc., Tokyo). Pots were placed in a thermostatic chamber at 20.0°C for 16 hr of daylight duration.

In July 2013, one individual plant was randomly chosen from each maternal plant offspring and transplanted to another pot (18.0 cm in diameter). This subset of plants was placed at a common garden in the campus of Shinshu University (36°15′N, 137°58′E, 650 m a.s.l.) in Matsumoto, Japan. The mean annual temperature was 11.8°C at Matsumoto Weather Station (610 m a.s.l.) between 1981 and 2010. The mean temperatures of August and January were 24.7 and −0.4°C, respectively. The annual mean precipitation was 1,031 mm, with most precipitation in summer. Although the elevation of our common garden was 650 m, it was ideal to set up common gardens at the same elevational range where samples were taken (1,597–2,779 m a.s.l.). However, as a practical problem, it was impossible to make such ideal common gardens, and we had to make a common garden in the university campus (650 m a.s.l.). However, the elevational differences in the phenological patterns of the *S. virgaurea* complex at the common garden reflected those in natural populations (see Results). Therefore, the common garden experiment at 650 m a.s.l. invalidates the results of this study.

The plants were watered once a day or once every 2 days, were fertilized once a week (N‐P‐K = 6‐10‐5, HYPONeX Japan Corp. Ltd., Osaka), and were relocated every week to reduce possible positioning effects during the first and second growing seasons. No bolting individuals appeared by the end of the first growing season (i.e., all plants remained as rosettes without flowers). In September 2013 (147th day after sowing), the rosette diameter in two perpendicular directions (one was the maximum width) was measured for each individual plant, and the above‐ground part was harvested. The rosette area was estimated as an ellipse. Each rosette leaf was separated into lamina and petiole, and the laminae of each individual plant were scanned. We measured the total lamina area of each plant using free software ImageJ 1.47 (http://rsbweb.nih.gov/ij/index.html). Laminae and petioles of each individual plant were weighed after oven‐drying at 80°C for 48 hr. The LMA of each individual plant was calculated as lamina dry mass divided by the lamina area. The above‐ground dry mass of each individual plant in the first growing season was calculated as the sum of the total lamina dry mass and petiole dry mass.

All laminae of each individual plant were ground into powder to measure nitrogen and chlorophyll concentrations. Leaf nitrogen concentration (%) was measured using FLASH2000 (Thermo Fisher Scientific Inc., Waltham). Leaf chlorophyll was extracted using dimethylformamide (4 ml). The absorbance of samples extracted from leaf samples at 663.8 and 646.8 nm was measured by using a spectrophotometer (UVmini‐1240, Shimadzu, Kyoto) and was substituted into Porra's equations (Porra, Thompson, & Kriedemann, 1989) to calculate chlorophyll *a* and *b* concentrations.

During the second growing season in May 2014, all survived individual plants were transplanted to new pots (25.0 cm in diameter) filled with potting compost. All individual plants formed scapes in the second growing season. Growth traits (i.e., stem height, below‐ground allocation (i.e., root mass), reproductive traits, and flowering phenology) were measured in the second growing season. We observed the presence or absence of flower buds, and we counted the number of flower heads and the number of flower heads that finished flowering for each individual plant every day. The number of flower heads per individual plant, the number of flower heads per day, and day of each phenological stage (onset day of bud formation, onset day of flowering, and finish day of flowering) were determined. Plants from the *S*. *virgaurea* complex form flower heads that are composed of ligulate flowers (female flower) and tubular flowers (bisexual flower). In the investigation of flowering phenology, the flower head was regarded as flowering when at least one tubular flower of the flower head was developed. Similarly, the flower head was regarded at the finish of flowering when all florets in a flower head had wilted and were discolored. We defined the following flowering phenological stages for each individual plant: (1) day of bud formation (the day when at least one bud appeared), (2) onset day of flowering (the day when at least one flower head flowered), (3) peak day of flowering (the day when most flower heads flowered), (4) finish day of flowering (the day when all flower heads had finished flowering). The flowering period was defined as between the onset and finish days of flowering for each individual. Ten flower heads were chosen randomly for all flowering individual plants, and their involucral length and diameter were measured by using a caliper.

Stem height was measured for all bolting individuals every week. The above‐ground part of each individual was harvested after the finish day of flowering. The above‐ground part of each individual was separated into stem, leaf, and reproductive organs, and each organ was stored in a separate envelope. Each organ was oven‐dried at 80°C for 48 hr and weighed. Seeds of the *S*. *virgaurea* complex tend to disperse from the plant by wind soon after seed maturation, and involucres also tend to drop from the plant after the release of seeds. The dispersed seeds and dropped involucres were not included in measuring plant biomass. Therefore, the sum of stem mass and leaf mass, except for seeds and involucres, was regarded as the above‐ground biomass in this study. The below‐ground parts (i.e., roots) were also dug up after the harvest of the above‐ground part. Soil on roots was washed out. The below‐ground biomass was weighed after oven‐drying at 80°C for 48 hr.

### 2.4 Molecular analyses

In June 2014 (second growing season of the common garden experiment), several leaves were sampled from each plant in the common garden for molecular analysis. In September 2014, 11–18 leaves of the *S*. *virgaurea* complex were additionally sampled from each of the nine study populations in the field. The leaf sample of each population was stored in an envelope and was kept at 3°C until DNA extraction.

Genomic DNA was extracted from a 5 mm square of leaf tissue using the DNeasy Plant Mini Kit (QIAGEN, Hilden, Germany). Sakaguchi and Ito (2014) and Sakata et al. (2013) developed microsatellite primers for the *S. virgaurea* complex and *Solidago altissima*, respectively. Here, 18 primers were selected (Sol_2003631, Sol_2005991, Sol_2006931, Sol_ 2007258, Sol_2012220, Sol_2013075, Sol_2071098, Sol_2001876, Sol_ 2003053, Sol_2005892, Sol_2007291, Sol_2007556, Sol_2013411, Sol_2015 992, Sol_2066912 from the *S. virgaurea* complex and salt1, salt3, salt17 from *S. altissima*). Sakaguchi and Ito (2014) and Sakata et al. (2013) describe details of primer pair sequences and molecular procedures. For all loci, the forward primer was synthesized with one of three different M13 sequences (5′‐CACGACGTTGTAAAACGAC‐3′, 5′‐TGTGGAATTGTGAGCGG‐3′, or 5′‐CTATAGGGCACGCGTGGT‐3′), and the reverse primer was tagged with a PIG‐tail sequence (5′‐GTTTCTT‐3′) to promote full adenylation (Brownstein, Carpten, & Smith, 1996).

PCR amplification was done in a 6 μl volume containing approximately 10 ng DNA, 2× Multiplex PCR Master Mix (QIAGEN, Hilden, Germany), 0.01 μmol/L of forward primer, 0.02 μmol/L of reverse primer, 0.1 μmol/L of M13 primer (fluorescently labeled with Beckman Dye, Beckman Coulter, Brea, California). The PCR thermal profile included denaturation at 94°C for 9 min, followed by 35 cycles of 94°C for 30 s, 57°C for 1 min, 68°C for 1 min, and a final 20‐min extension step at 68°C. The PCR products (1 μl) were separated by electrophoresis by using an automated sequencer (CEQ 8000 Genetic Analysis System, Beckman Coulter Brea, California) with 0.4 μl of CEQ DNA Size Standard‐600 (Beckman Coulter, Brea, California) and 20 μl of Sample Loading Solution (Beckman Coulter, Brea, California). The fragment size and allele identification were determined using software Fragment Analysis version 8.0 (Beckman Coulter Brea, California).

### 2.5 Data analysis

#### 2.5.1 Common garden experiment

A generalized linear mixed model (GLMM) was used to analyze morphological and physiological traits and flowering phenology in the common garden among the nine populations with different elevations of provenance sites at which seeds were originally collected. The germination rate per population and mean seed mass per maternal plant were regressed against the elevation of provenance sites by GLMM. Maternal plants were treated as a random effect because the germination rate and seed mass may differ between maternal plants due to genetic differences, even at the same elevation. The GLMM was also used to analyze relationships of the elevation of provenance sites with morphological and physiological traits, that is, stem height, above‐ground biomass, below‐ground biomass, rosette area, ratio of below‐ground biomass to total biomass (above‐ and below‐ground biomass), LMA, leaf nitrogen and chlorophyll *a *+ *b* concentrations, chlorophyll *a*/*b* ratio, number of flower heads per individual plant. Relationships of four phenological stages (day of bud formation, onset, peak, and finish day of flowering) against the elevation of provenance sites were also analyzed using GLMM. Statistical significance of each regression model was assessed using a likelihood ratio test. Distribution of likelihood ratio in a null model was computed as the chi‐square distribution approximation.

Mean involucre length and diameter were compared among the nine elevations of provenance sites using the nonparametric Kruskal–Wallis test. The nonparametric Steel–Dwass multiple comparison test was used to compare each pair of populations.

#### 2.5.2 Population genetic analyses

To assess the population genetic structure and the gene flow among the nine populations of the *S*. *virgaurea* complex along an elevational gradient, we analyzed the genotyping data obtained with the 18 microsatellite markers. Deviation from the Hardy–Weinberg equilibrium was estimated within a population by using the probability test for Genepop 4.4.3 (Raymond & Rousset, 1995). Deviations from Hardy–Weinberg equilibrium were also assessed per locus by using the exact test. Linkage disequilibrium was determined using Fisher's exact test in Genepop 4.4.3 (Raymond & Rousset, 1995).

Bayescan 2.1 (Foll & Gaggiotti, 2008) was used to detect candidate outlier loci, because such loci that departed from neutral evolution (outlier loci) can bias estimates of population dynamics. In the Bayescan analysis, the algorithm divides genetic differentiation into elements specific to the locus (α) and the element of differentiation among populations (β) and then detects the outlier locus following non‐neutral evolution.

Number of alleles (*A*), effective number of alleles (*A*_E_), observed heterozygosity (*H*_O_), expected heterozygosity (*H*_E_) were calculated by GenAlEx 6.502 (Peakall & Smouse, 2006). Allelic richness (*A*_R_: El Mousadik & Petit, 1996) per locus in each population was calculated using FSTAT 2.9.3.2 (Goudet, 1995), and then, the mean value and standard error were calculated. One‐way ANOVA evaluated whether *A*_R_ differs between the nine populations.

Genetic differentiation between populations was estimated, based on the coefficient of *F*_ST_. Negative *F*_ST_ values were converted into zero in this study. Pairwise *F*_ST_ values between each pair of populations were computed using Arlequin 3.5.2.2 (Excoffier, Laval, & Schneider, 2005). Analysis of molecular variance (AMOVA) was used to partition the genetic variance among populations using Arlequin 3.5.2.2 (Excoffier et al., 2005). Principal coordinate analysis (PCoA) was also conducted to detect genetic differentiation among the nine populations using GenAlEx 6.502 (Peakall & Smouse, 2006).

The geographic distance between each pair of populations was calculated from the latitude and longitude of sampling sites. Similarly, the elevational distance between each pair of populations was calculated. We examined correlations of genetic differentiation (*F*_ST_) with geographic distances and with elevational distances using the Mantel test. A partial Mantel test was used because the geographic distance correlated to the elevational distance in this study. All statistical analyses were conducted using the free software R version 3.1.2 (R Core Team, 2015), and the “vegan” package (Oksanen et al., 2013) was used for the Mantel test and partial Mantel test.

## 3 RESULTS

### 3.1 Germination experiment

**Figure 1:**
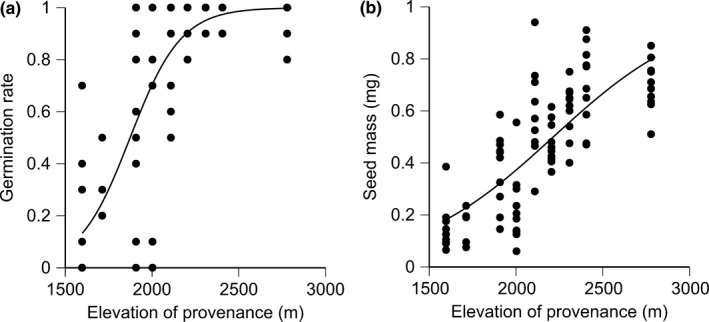
Relationships of elevation of provenance sites with (a) germination rate and (b) seed mass of the *Solidago virgaurea* complex. Table 2 shows the results of generalized linear mixed model

The germination rate showed a sigmoid curve against elevations of provenance sites at which the seeds were originally collected (Figure 1a, Table 2, *p* < .001). Seed mass was also greater for seeds of higher elevations of provenance sites (Figure 1b, *p* < .01).

**Table 2:** Results of generalized linear mixed models for seed traits and various plant traits in the common garden experiment. Each response variable was regressed against the elevation of provenance (predictor variable). The significance of regression was examined by the Wald test

Response variable	Model (*Y *= *aX *+ *b*)					Pr (Chi)
	*n*	Probability distribution	Link function	Slope *a*	Intercept *b*	
Seed						
Germination rate	85	Binomial	Logit	0.006808	−12.753	<0.001
Seed mass (mg)	85	Binomial	Logit	0.002431	−5.384	<0.01
Common garden experiment						
First growing season						
Rosette area (cm^2^)	55	Gamma	Log	−0.000466	6.560	0.0877
Above‐ground biomass (g)	56	Gamma	Log	−0.000590	1.847	<0.05
Leaf mass per area (g m^−2^)	56	Gamma	Log	−0.000139	4.519	<0.001
Nitrogen concentration (%)	55	Gamma	Log	0.000244	0.186	<0.001
Chlorophyll concentration (%)	55	Gamma	Log	0.000221	−2.169	<0.001
Chlorophyll *a*/*b* ratio	55	Gamma	Log	0.000215	0.259	<0.01
Second growing season						
Stem height (cm)	31	Gamma	Log	−0.001520	6.749	<0.001
Above‐ground biomass (g)	41	Gamma	Log	−0.001485	5.525	<0.001
Below‐ground biomass (g)	21	Gamma	Log	−0.000214	2.454	0.426
Ratio of below‐ground biomass to total biomass	21	Gamma	Log	0.000764	−2.704	<0.001
Number of flower heads per individual	31	Poisson	Log	−0.004193	15.490	<0.001
Flowering phenology						
Day of bud formation	31	Poisson	Log	−0.000516	6.013	<0.001
Onset day of flowering	31	Poisson	Log	−0.000593	6.308	<0.001
Peak day of flowering	31	Poisson	Log	−0.000734	6.772	<0.001
Finish day of flowering	31	Poisson	Log	−0.000570	6.650	<0.001
Flowering period	31	Poisson	Log	−0.000532	5.421	<0.05

### 3.2 Common garden experiment

**Figure 2:**
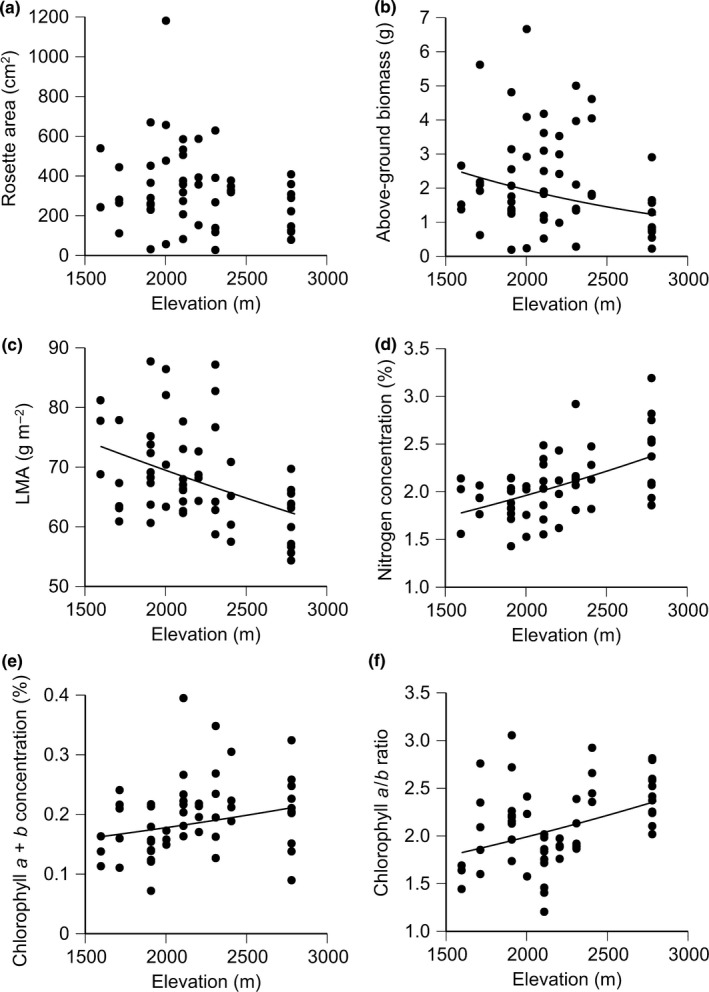
Relationships of elevation of provenance sites with (a) rosette area, (b) above‐ground biomass, (c) leaf mass per area (LMA), (d) nitrogen concentration, (e) chlorophyll *a*+*b* concentration, (f) chlorophyll *a*/*b* ratio of the *Solidago virgaurea* complex in the first growing season of the common garden experiment. Table 2 shows the results of generalized linear mixed model. A regression line is not shown in graph (a) because of no statistical significance (*p *>* *.05)

In the first growing season, rosette area did not correlate with the elevation of provenance sites (Figure 2a, Table 2). However, above‐ground biomass (Figure 2b, *p* < .05) and LMA (Figure 2c, *p* < .05) were significantly lower for higher elevations of provenance sites. Leaf nitrogen, chlorophyll *a*+*b* concentrations, and chlorophyll *a*/*b* ratio significantly increased with elevation of provenance sites (at least *p *<* *.01, Figure 2d–f).

**Figure 3:**
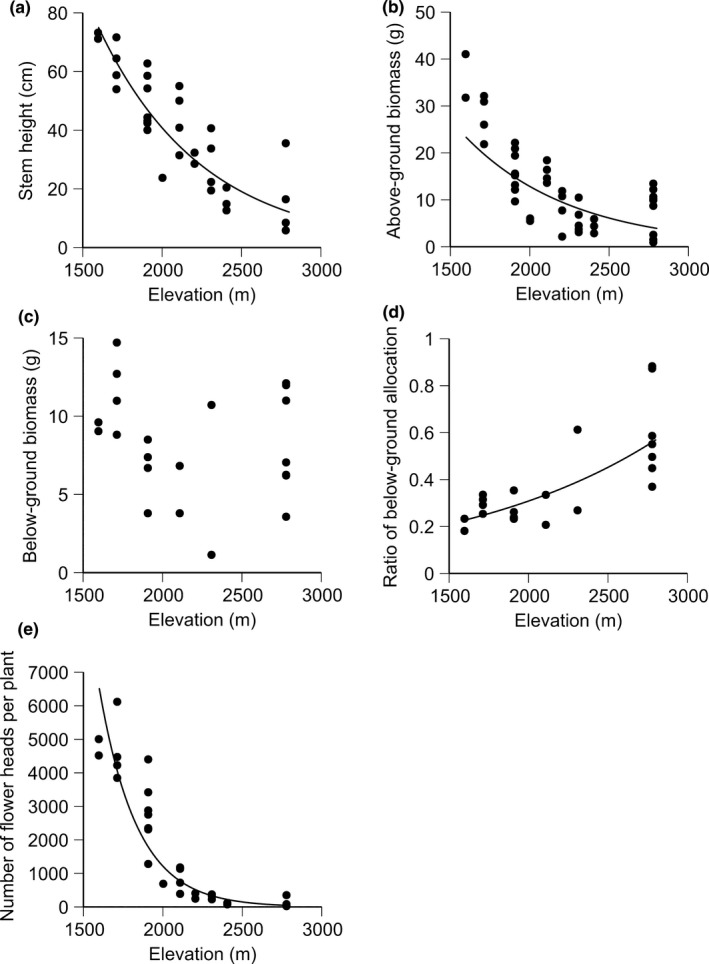
Relationships of elevation of provenance sites with (a) stem height, (b) above‐ground biomass, (c) below‐ground biomass, (d) below‐ground allocation (%) (proportion of below‐ground biomass to the total biomass), (e) number of flower heads per individual of the *Solidago virgaurea* complex in the second growing season of the common garden experiment. Table 2 shows the results of generalized linear mixed model. A regression line is not shown in the graph (c) because of no statistical significance (*p *>* *.05)

In the second growing season, stem height and above‐ground biomass significantly decreased with elevation of provenance sites (*p *<* *.001, Figure 3a,b, Table 2). Below‐ground biomass did not correlate with elevation of provenance sites (Figure 3c), and so the proportion of below‐ground biomass in the total biomass significantly increased with elevation of provenance sites (*p *<* *.001, Figure 3d). The number of flower heads per individual significantly decreased with elevation of provenance sites (*p *<* *.001, Figure 3e).

**Figure 4:**
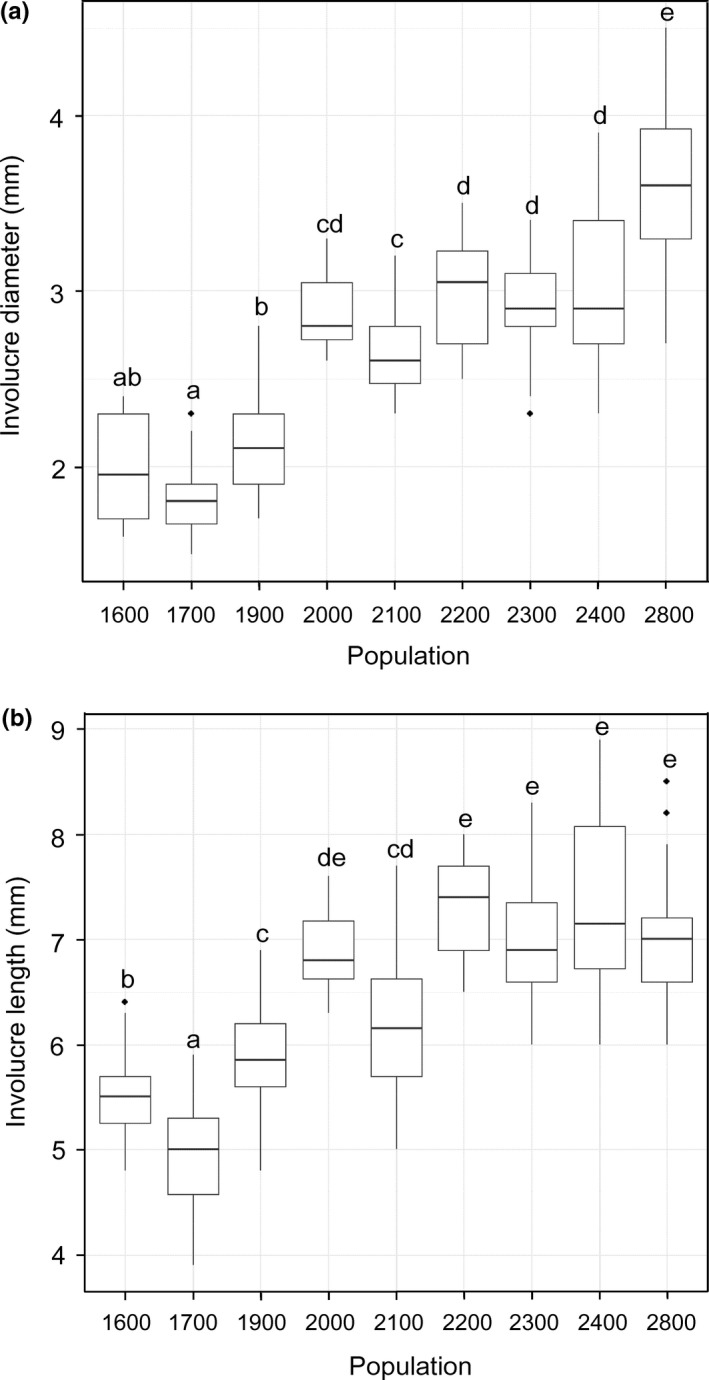
Boxplots of (a) involucral diameter and (b) involucral length of the *Solidago virgaurea* complex in the second growing season of the common garden experiment. The bottom and top of each box are the first and third quartiles, respectively, and the band inside the box is the median. The length of whiskers extending from the lower and upper quartiles is the 1.5× the interquartile range. Data outside both ends of whiskers are outliers and plotted as individual points. The same alphabet letter indicates no significant difference between populations at *p *=* *.05 by the Steel–Dwass Test

**Figure 5:**
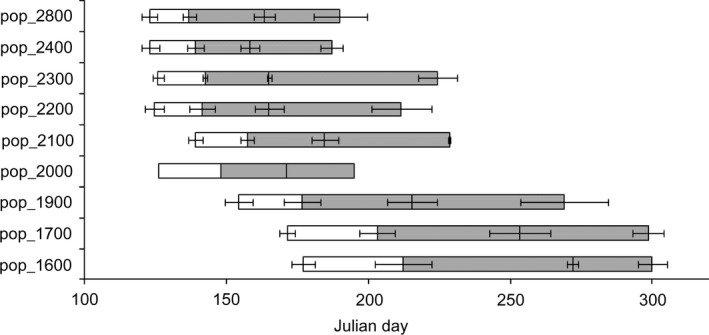
Differences of flowering phenology among the nine populations in the second growing season of the common garden experiment. Shaded areas indicate the flowering period. Open areas indicate periods of bud formation. The left and right parts of shaded areas indicate onset days and finish days of flowering, respectively. The vertical line between the left and right parts of the shaded area indicates the peak day of flowering for each population. Four horizontal lines are standard errors of day of bud formation, onset, peak, and finish days of flowering from left to right, respectively, for each population. The standard error is not shown for pop_2000 because only one individual formed flower heads

Involucral diameter and length were greater for high elevations of provenance sites (2,000 m to 2,800 m a.s.l.) than for low elevations (1,600 m to 1,900 m a.s.l.) (Figure 4). The flowering phenological stages (day of bud formation, onset, peak, and finish day of flowering) started earlier at higher elevations of provenance sites (Figure 5), and flowering period was also shorter there.

### 3.3 Genetic structure and gene flow

Two loci deviated from the Hardy–Weinberg equilibrium in more than half of the populations (six populations for Sol_2003631 and eight populations for Sol_2005892). These two loci were removed from further analyses because they likely harbor null alleles. The Bayescan showed that loci Sol_2015992 and Sol_2066912 of the 16 loci deviated significantly from neutral evolution (*p *<* *.05). However, these loci were not excluded from the further analyses because results did not change after their removal.

**Figure 6:**
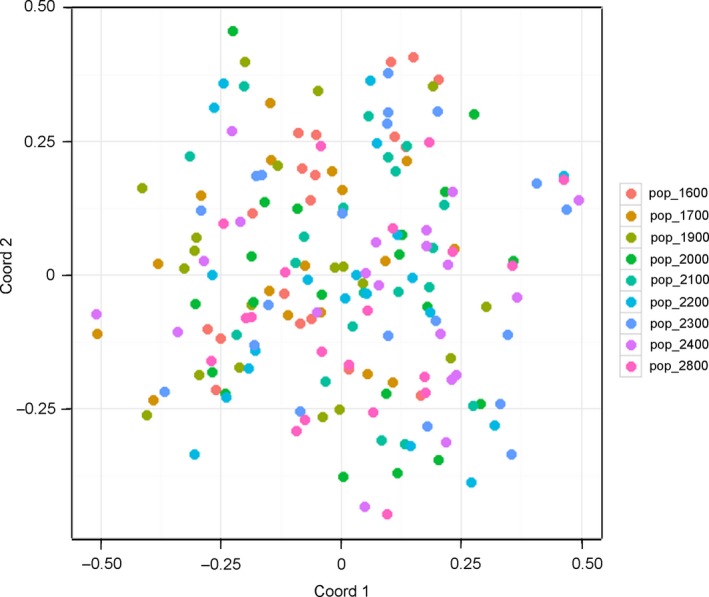
Result of principal coordinate analysis (PCoA) using genotype data obtained by 16 microsatellite markers in the *Solidago virgaurea* complex. Each datum shows one individual. Each population showed no clusters

Analysis of the 16 microsatellite loci showed that the number of alleles per population (*A*) was 4.44–5.00, the effective number of alleles per population (*A*_E_) was 2.24–2.57, the observed heterozygosity (*H*_O_) was 0.38–0.46, and the expected heterozygosity (*H*_E_) was 0.44–0.48 (Table 3). The mean value of *A*_R_ per population was 4.38–4.74, and the result of ANOVA indicated that the genetic diversity did not differ significantly among the nine populations (Table 3, *p* = .964). The range of pairwise *F*_ST_ values was considerably narrow (between 0 and 0.027). Of the 38 pairs, only 10 pairs showed *F*_ST_ values significantly different from zero (Table 4). Most genetic variation was partitioned into individuals within a population, and the genetic differentiation among populations was not significant, based on AMOVA (Table 5, *p* = 1.000, *F*_ST_ = 0.005). The principal coordinate analysis also showed no genetic differentiation among the nine populations, that is, individuals from different elevations forming one group (Figure 6).

**Table 3:** Genetic variations of the *Solidago virgaurea* complex at 16 microsatellite loci. Mean values with standard error are shown for each variable of each population

Population	*n*	*A*	*A*_E_	*A*_R_	*H*_O_	*H*_E_
pop_1600	19	4.56 ± 0.61	2.37 ± 0.34	4.43 ± 0.58	0.42 ± 0.07	0.46 ± 0.07
pop_1700	17	4.44 ± 0.62	2.24 ± 0.30	4.44 ± 0.62	0.39 ± 0.07	0.44 ± 0.07
pop_1900	19	4.69 ± 0.64	2.50 ± 0.44	4.57 ± 0.62	0.42 ± 0.07	0.45 ± 0.07
pop_2000	20	4.56 ± 0.56	2.57 ± 0.46	4.39 ± 0.54	0.44 ± 0.07	0.46 ± 0.07
pop_2100	20	5.00 ± 0.65	2.55 ± 0.43	4.74 ± 0.61	0.46 ± 0.07	0.48 ± 0.07
pop_2200	20	4.94 ± 0.57	2.49 ± 0.37	4.70 ± 0.54	0.45 ± 0.07	0.48 ± 0.07
pop_2300	20	4.56 ± 0.57	2.37 ± 0.38	4.38 ± 0.55	0.38 ± 0.06	0.45 ± 0.07
pop_2400	20	4.63 ± 0.48	2.42 ± 0.35	4.43 ± 0.47	0.42 ± 0.07	0.47 ± 0.07
pop_2800	20	4.75 ± 0.51	2.49 ± 0.36	4.51 ± 0.49	0.45 ± 0.06	0.48 ± 0.07

*N*, total number of samples from the common garden experiment and field; *A*, number of alleles; *A*_E_, effective number of alleles; *H*_O_, observed heterozygosity; *H*_E_, expected heterozygosity; *A*_R_, allelic richness.

**Table 4:** Pairwise *F*_ST_ values between the *Solidago virgaurea* complex populations

	pop_1600	pop_1700	pop_1900	pop_2000	pop_2100	pop_2200	pop_2300	pop_2400	pop_2800
pop_1600									
pop_1700	0.018^a^								
pop_1900	0.017^a^	0.000							
pop_2000	0.019^a^	0.008	0.009						
pop_2100	0.026^a^	0.017^a^	0.013^a^	0.001					
pop_2200	0.023^a^	0.013	0.001	0.001	0.000				
pop_2300	0.021^a^	0.006	0.015	0.000	0.002	0.002			
pop_2400	0.009	0.008	0.006	0.000	0.000	0.000	0.000		
pop_2800	0.027^a^	0.021^a^	0.009	0.005	0.000	0.000	0.003	0.000	

a *p *<* *.05.

**Table 5:** Results of AMOVA for the *Solidago virgaurea* complex among populations, among individuals within population, within individuals

Source of variation	*df*	Sum of squares	Variance components	Percentage of variation	Fixation indices
Among populations	8	37.749	0.01850	0.5	*F*_ST_ = 0.005
Among individuals within a population	166	663.914	0.28831	7.73	*F*_IS_ = 0.078^a^
Within individuals	175	599.000	3.42286	91.77	*F*_IT_ = 0.082^a^
Total	349	1,300.663	3.72967		

a *p *<* *.001.

**Figure 7:**
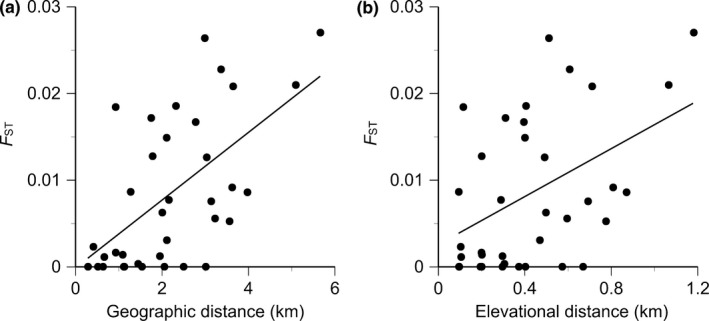
Relationships of *F*_ST_ values of the *Solidago virgaurea* complex with (a) geographic distance (*r *=* *.61, Mantel‐*p *=* *.003) and (b) elevational distance (*r *=* *.47, Mantel‐*p *=* *.047)

The genetic differentiation between populations (pairwise *F*_ST_) was correlated significantly with geographic distances (*r *=* *.61, Mantel‐*p *=* *.003, Figure 7a) and elevational distances (*r *=* *.47, Mantel‐*p *=* *.047, Figure 7b). The pattern of isolation by geographic distance was found even if the partial Mantel test controls for the effect of elevation (*r *=* *.65, Mantel‐*p *=* *.001). However, the pattern of isolation by elevation was not found if the partial Mantel test regulates the effect of the geographic distance (*r *= −.63, Mantel‐*p *=* *.999). Therefore, these results indicate that the significant correlation between the elevational distance and pairwise *F*_ST_ values may be caused by high correlation between geographic distance and elevational distance.

## 4 DISCUSSION

### 4.1 Seed size and maternal effects

Seeds of studied plants of the *S*. *virgaurea* complex from higher elevations showed greater seed mass and germination rates than those from lower elevations. A trade‐off relationship exists between the number of seeds and seed mass (Primack, 1978). Species with smaller seeds produce a larger number of seeds and disperse small seeds in a wide area, which contributes to avoidance of sibling competition (Augspurger, 1984; Howe & Richer, 1982). The germination rate and seedling establishment are greater for species with larger seeds (Westoby, Leishman, & Lord, 1996). Some studies showed the increase of seed size at high elevations with severe environmental conditions for seedling establishment because of a large storage of carbohydrate (Baker, 1972; Mariko, Koizumi, Suzuki, & Furukawa, 1993; Moles et al., 2007; Pluess, Schütz, & Stöcklin, 2005). Thus, the strategy to increase seed germination and seedling establishment is thought to be advantageous at high elevations with a short growing season.

Possibly, the phenotypic variation measured in the common garden experiment partly reflected the environmental maternal effect because all seeds used were obtained from natural populations (Monty, Lebeau, Meerts, & Mahy, 2009). Growth environments of maternal plants influence phenotypes of their offsprings through morphological and physiological plasticities of maternal plants (Roach & Wulff, 1987). For example, environmental conditions of zygotes often affect phenotypes of the sporophyte (Kirkpatrick & Lande, 1989; Schmid & Dolt, 1994). These environmental maternal effects are often transmitted to offsprings through seed mass or size. Growth environments of maternal plants also affect the seed size; the seed size positively correlates with the germination rate and the following plant growth (Schmid & Dolt, 1994; Weis, 1982). However, the environmental maternal effect is marked in the early stages of the life history only and generally decreases with progress of individual growth (Schmid & Dolt, 1994; Wulff & Bazzaz, 1992). Therefore, phenotypic variations measured in the common garden experiment over 2 years are suggested to be caused by genetic differentiation rather than by the environmental maternal effect.

### 4.2 Ecological interpretation of phenotypic variation in the common garden experiment

In the first growing season of the common garden experiment, rosette area did not correlate with elevation of provenance sites. However, above‐ground biomass and LMA decreased, and leaf nitrogen and chlorophyll concentrations increased with increasing elevation of provenance sites. These observed patterns corresponded to those related to populations of the *S*. *virgaurea* complex along an elevational gradient in the field (Takahashi & Matsuki, 2017; our unpublished data). Leaf nitrogen concentration positively correlates with the light‐saturated maximum photosynthetic rate (Reich, Walters, Ellsworth, & Uhl, 1994). The increase in chlorophyll *a*/*b* ratio indicates that there is more chlorophyll *a* having a higher light condensing capability than chlorophyll *b* that is the reaction center chlorophyll and is regarded as an adaptation to increase the photosynthesis rate in high light conditions (Peng, Wu, Xu, & Yang, 2012). The *S*. *virgaurea* complex is a deciduous perennial herb species, and the stem and leaves wither before winter. Therefore, the positive carbon balance at the level of individual leaves can be maintained by decreasing the leaf structural cost and increasing the maximum photosynthetic rate at high elevations with a shorter growing season (Kudo, 1996, 1999; Oleksyn et al., 1998).

In the second growing season of the common garden experiment, the stem height and the above‐ground biomass decreased with an increase in elevation of provenance sites. These variation patterns corresponded to those observed in the field (Takahashi & Matsuki, 2017) because of resource limitation due to severe environmental conditions, that is, short growing season, prolonged snow cover, strong wind, and shallow soil (Clausen et al., 1948; Körner, 2003; Mizuno, 1991; Natori, 1964; Takahashi & Yoshida, 2009). The proportion of below‐ground biomass in the total biomass increased with elevation of provenance sites. Natori (1964) and Shibata, Kinoshita, and Arai (1975) also reported higher ratios of below‐ground biomass to above‐ground biomass in the *S*. *virgaurea* complex at higher elevations. Probably, the *S*. *virgaurea* complex reserves photosynthate in the below‐ground part to grow soon after the start of the next growing season at high elevations with a short growing period.

Although some morphological traits, such as LMA and stem height, continuously changed with elevation of provenance sites in the common garden experiment, the morphology of involucre discontinuously changed at the boundary between the distribution areas of the two subspecies (1,950 m a.s.l.). Flowers of the *S*. *virgaurea* complex are entomophilous (Sakurai & Takahashi, 2017). However, the number of pollinators decreases at higher elevations with cooler conditions (Blionis, Halley, & Vokou, 2001; Maad, Armbruster, & Fenster, 2013). Size of flower heads of the *S. virgaurea* complex is larger in higher elevations (Nishizawa et al., 2001; Takahashi & Matsuki, 2017). Large flower heads may increase the reproductive success by increasing the chance to attract pollinators (Brody & Mitchell, 1997).

### 4.3 Elevational differences of flowering phenology

Each stage of flowering phenology started earlier for plants at higher elevations of provenance sites, indicating that flowering phenology differs along the elevational gradient. This result corresponds to previous studies (Sakurai & Takahashi, 2017; Shibata & Terauchi, 1990) in which also reported earlier flowering of the *S*. *virgaurea* complex at higher elevations in the field. Early flowering is adaptive to high elevations because plants must produce mature seeds by the end of the short growing period (Sandring, Riihimäki, Savolainen, & Agren, 2007). In contrast, the *S*. *virgaurea* complex plants from lower elevations of provenance sites grew for longer periods and flowering started later. The increase in plant height due to long growth periods at lower elevations would be advantageous not only for competition with other plants, but also for reproduction because the increase in plant height increases the number of flower heads per individual in the *S. virgaurea* complex (Kiełtyk & Mirek, 2014; Takahashi & Matsuki, 2017).

Assuming the time of flowering is regulated by the effective accumulated temperature, *S*. *virgaurea* populations at higher elevational ranges are considered to start flowering genetically with a lower effective accumulated temperature than populations at lower elevations. In our study, flowering of individuals grown in the common garden (650 m a.s.l) started some months earlier than natural populations of the *S. virgaurea* complex in the subalpine zone (1,597 to 2,779 m a.s.l.), suggesting that plants grown in the common garden attained the effective accumulated temperature necessary to flower earlier than natural populations in the subalpine zone because the common garden location was warmer than the subalpine zone. Therefore, the elevational differences in phenological patterns in the common garden reflected those in natural populations; nevertheless, there were elevational differences between the common garden and natural populations of the *S. virgaurea* complex.

### 4.4 Local adaptation under existence of gene flow

Although neutral loci had only very low genetic differentiation among the nine populations, many morphological and physiological traits genetically differed among the nine populations at the common garden. Each population showed a high genetic diversity, suggesting that stochastic fluctuation of population size has not been dominant. Populations would rather have been stable along the elevational gradient and connected genetically under strong gene flow, as promoted by pollen dispersal by various insects, such as hoverflies and butterflies, and seed dispersal by anemochory (Kawano, 1988; Sakurai & Takahashi, 2017). It is possible that genetic bases of plant traits are genetically different at different elevations in the field even under gene flow, if strong selection pressure operates along environmental gradients (Gonzalo‐Turpin & Hazard, 2009; Hall et al., 2007; Sambatti & Rice, 2006). Alleles of genes of adaptive traits possibly differ among populations, even if molecular analysis using neutral markers cannot detect population differentiation (Bakessy, Ennos, Burgman, Newton, & Ades, 2003). For example, *Arabidopsis halleri* subsp. *gemmifera* shows elevational phenotypic variation, like the *S. virgaurea* complex, and clinal changes have been detected in adaptive gene alleles in its populations along elevational gradients under gene flow (Kubota et al., 2015).

Although flowering of the *S*. *virgaurea* complex starts approximately 2 weeks later at elevations below 400 m, flowering lasts more than 1 month at each elevation (Sakurai & Takahashi, 2017). Thus, gene flow is presumed to occur between neighboring elevations, following the stepping stone model of population structure (Kimura & Weiss, 1964). The detected isolation by geographic distance suggests that gene flow and immigration continuously occur between neighboring elevations because the pairwise *F*_ST_ values were very low. Therefore, while most parts of neutral genomic regions of the *S. virgaurea* complex populations are homogenized by active gene flow, particular regions linked to adaptive traits are suggested to be differentiated by strong selection pressure (i.e., shorter growing season in higher elevations), which in turn causes phenotypic variations observed in the common garden experiment.

## 5 CONCLUSION

In this study, *S*. *virgaurea* complex populations along an elevational gradient were shown to be linked by substantial gene flow between neighboring populations by molecular analysis using neutral microsatellite markers. However, many morphological and physiological traits and flowering phenology showed genetic differentiation along elevations of provenance sites in the common garden experiment. These results suggest that only the genome regions of adaptive traits may display differentiation due to strong selection pressures despite the existence of gene flow. Our findings provide an example of plant micro evolution that genetically maintains adaptive traits to their local environments, even in narrow geographical ranges and under gene flow that could homogenize local adaptation.

In our study area (Mt. Norikura), *S*. *virgaurea* subsp. *asiatica* and *S. virgaurea* subsp. *leiocarpa* are distributed below and above ca. 1,950 m a.s.l., respectively, based on morphological characteristics of flower heads (Nishizawa et al., 2001). However, the molecular analysis provided by study, using 16 microsatellite markers, did not support the differentiation of the *S*. *virgaurea* complex into the two subspecies. Likewise, Nakamura et al. (1997) reported no genetic differentiation between the two subspecies on Mt. Hakusan in central Japan, using the RAPD and FISH methods. Considering their result together with our findings, it is plausible that the two subspecies are not genetically differentiated from each other, at least in terms of neutral loci. Therefore, the widespread alpine taxon of *S. virgaurea* subsp. *leiocarpa* would be an ecotypic entity that arose from ancestral species via ecological adaptation to alpine environments. However, more comprehensive genetic analysis of the two subspecies (or ecotypes) in multiple mountain ranges is needed to understand the origins of alpine subspecies of subsp. *leiocarpa* in Japan.

## CONFLICT OF INTEREST

None declared.
